# Mapping the Global Trajectory and Key Trends of Temporal Interference Stimulation

**DOI:** 10.3390/bioengineering13070741

**Published:** 2026-06-25

**Authors:** Li Qi, Zhishun Gao, Xiaomin Pan, Jin Li, Yue Yu, Kai Wang, Qianqian Li, Tongjian Bai

**Affiliations:** 1 Department of Neurology, The First Affiliated Hospital of Anhui Medical University, Hefei 230022, China; 2 Department of Psychology and Sleep Medicine, The Second Affiliated Hospital of Anhui Medical University, Hefei 230601, China; 3 The School of Mental Health and Psychological Sciences, Anhui Medical University, Hefei 230032, China; 4 Institute of Artificial Intelligence, Hefei Comprehensive National Science Center, Hefei 230088, China; 5 Anhui Province Key Laboratory of Cognition and Neuropsychiatric Disorders, Hefei 230032, China; 6 Collaborative Innovation Center of Neuropsychiatric Disorders and Mental Health, Hefei 230032, China; 7 Anhui Institute of Translational Medicine, Hefei 230032, China

**Keywords:** temporal interference stimulation, neural engineering, biophysical modeling, electric field dosimetry, optimization strategies

## Abstract

Since its inception in 2017, temporal interference stimulation (TIS) has attracted increasing attention as a novel neuromodulation approach with the potential to non-invasively target deep brain structures. As the field moves from initial biophysical validation toward broader experimental and translational applications, a macroscopic understanding of its developmental trajectory and thematic evolution is needed. In this study, we systematically mapped the scientific landscape of TIS research using bibliometric methods to characterize its knowledge structure, core themes, and emerging frontiers. The analysis shows that TIS research has expanded rapidly from foundational animal studies and biophysical mechanism validation toward computational head modeling, individualized electric field optimization, and early human applications. Current research is increasingly focused on cross-species scaling, stimulation dosimetry, comparative advantages over other neuromodulation techniques, precise targeting strategies, and potential physiological risks such as high-frequency conduction block. Overall, TIS is evolving from an exploratory biophysical concept into a promising but technically and physiologically complex neuromodulation tool. Overcoming current engineering and translational barriers, particularly through individualized modeling, rigorous optimization, and well-designed human studies, will be essential for establishing TIS as a reliable therapeutic intervention.

## 1. Introduction

The modulation of deep brain structures is a rapidly developing therapeutic option for many neurological and psychiatric disorders [1], as these regions are critical nodes in neural circuits associated with conditions such as Parkinson’s disease [2], dystonia, tremor [3], and epilepsy [4,5]. For decades, the primary approach to accessing deep brain structures has been Deep Brain Stimulation (DBS), an invasive method involving the surgical implantation of electrodes to deliver electrical stimulation directly to target nuclei [6,7]. However, the invasive nature of DBS carries inherent risks, such as surgical complications and infection, and the fixed location of the implant limits the ability to change the stimulation area post-implantation [8].

In contrast, Non-invasive Brain Stimulation (NIBS) techniques are important neuromodulation modalities [9], with common methods including Transcranial Magnetic Stimulation (TMS) and Transcranial Electrical Stimulation (tES) [10]. These non-invasive techniques offer the advantages of being safe, tolerable, and easy to administer [11]. By applying electrical or magnetic fields to the scalp, these methods induce acute and neuroplastic changes in cortical excitability [12,13] and have received FDA approval for treating conditions such as depression and obsessive compulsive disorder [14]. However, a major limitation of conventional NIBS is its lack of focal depth. Due to the complex structure of the human brain, magnetic and electrical signals are absorbed and scattered within brain tissue, and the fields typically decrease dramatically with depth [15,16]. As a result, these techniques primarily modulate superficial cortical areas and have not been proven to directly modulate deep brain neuron activity without affecting the overlying cortex [17]. Despite advances such as Deep TMS (dTMS) and Transcranial Focused Ultrasound (TFU) for deeper targets, both remain limited, with dTMS constrained by spatial precision and TFU by resolution and safety concerns related to skull energy absorption [18,19].

Temporal Interference Stimulation (TIS) was introduced in 2017 to overcome these challenges [20]. The principle of TIS is based on the low-pass filtering properties of the neuronal membrane, which means neurons may not respond to high-frequency oscillating (e.g., ≥1 kHz) electric fields [21]. TIS is based on the simultaneous application of at least two high-frequency (≥1 kHz) currents with slightly different frequencies (e.g., 2 kHz and 2.01 kHz) [22]. At the location where the electric fields intersect, they interfere to create an amplitude-modulated field with a low-frequency “envelope” or “beat” that oscillates at the difference between the two carrier frequencies (e.g., 10 Hz) [23]. This low-frequency envelope is proposed to modulate deep-lying neurons while minimizing recruitment of overlying regions, which are primarily exposed to high-frequency fields [20,21].

Since its conception, TIS has garnered significant global attention because of its potential to non-invasively modulate deep brain regions, which may have implications for the treatment of neurological disorders [24]. As a nascent technology, it has spurred a body of research spanning computational models, animal studies, and human trials [25]. Given this rapid emergence and expanding interest, a comprehensive and quantitative analysis of the existing literature is needed to understand the structure and trajectory of this new field. Bibliometric analysis provides a powerful tool to systematically map the scientific landscape, identify core knowledge structures, pinpoint research hotspots, and track evolving trends [26]. Therefore, this study employs bibliometric methods to provide a macroscopic overview of TIS research, offering a valuable reference for understanding its current state and guiding future investigations. Given the early stage of the field, synonymous terms appear inconsistently across published literature. Throughout this manuscript, TIS is used as the standard umbrella term for the non-invasive deep brain neuromodulation technique.

## 2. Material and Methods

### 2.1. Data Collection and Search Strategy

The bibliographic data analyzed in this study were retrieved from the Web of Science Core Collection (WoSCC) database on 1 October 2025. WoSCC was selected because it provides standardized bibliographic metadata and structured cited-reference information required for co-citation, citation burst, collaboration network, and keyword analyses, and its export format is compatible with bibliometrix, VOSviewer, and CiteSpace. The use of a single primary database also avoided inconsistencies in citation counts, author names, institutional affiliations, and cited-reference formats that may arise when records from multiple databases are directly merged. No time restriction was applied to the publication years. The search was conducted using keywords related to temporal interference stimulation, such as “temporal interference”, “temporally interfering”, “stimulation”, and “electric”. After removing duplicate records, a total of 181 publications were included in the final dataset. Complete records for these publications, including cited references, were exported as plain text files for subsequent analysis.

### 2.2. Data Analysis

A comprehensive bibliometric analysis was conducted using multiple software tools to ensure analytical rigor and visualization clarity. Statistical analyses and the generation of publication trend plots and citation distributions were performed in R software (version 4.3.2) with the bibliometrix package (version 5.1.1, http://www.bibliometrix.org) (accessed on 23 June 2026) [27]. Bibliometrix was additionally used to identify the most locally cited authors and the most relevant affiliations. Local citations were defined as citations received from other publications within the included dataset, whereas affiliations were ranked according to their associated publication output. VOSviewer (version 1.6.20, https://www.vosviewer.com/) ((accessed on 23 June 2026) and CiteSpace (version 6.3.R1) [28,29] were employed to construct and visualize scientific knowledge maps. To ensure data reliability, two authors independently extracted and cross-validated the bibliometric data. VOSviewer was used to generate and visualize co-authorship networks of countries and institutions, co-citation networks of sources, and keyword co-occurrence networks. The inclusion thresholds were set as follows: a minimum of two documents per country or institution for co-authorship analysis, at least twenty citations per source for co-citation analysis, and a minimum of five occurrences per keyword for co-occurrence analysis. Before keyword co-occurrence analysis, targeted thesaurus-based standardization was applied to core TIS-related terms and spelling variants, including terms referring to “temporal interference”, and “temporally interfering”, to reduce fragmentation of the central topic term. Citation bursts were detected using CiteSpace to identify references exhibiting an unusually rapid increase in citation frequency during a specific period. References were ranked according to burst strength across the complete observation period. Journal Impact Factors (IFs) for 2024 were obtained from the 2025 edition of the Journal Citation Reports (JCR).

## 3. Results

### 3.1. Overview of Publication Trends and Leading Contributors

**Figure 1:**
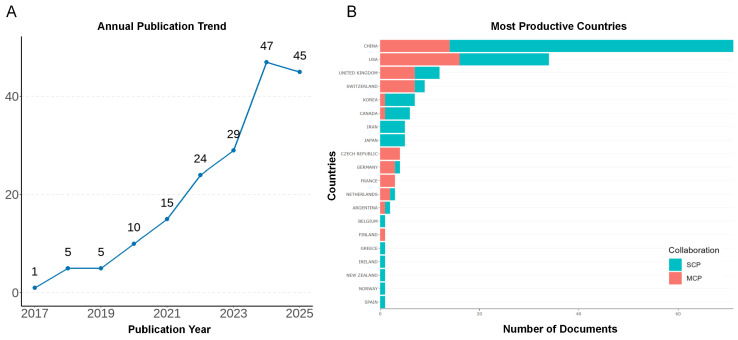
Annual publication trends and country contributions in TIS research based on data retrieved from the Web of Science Core Collection as of October 1, 2025. (**A**) Annual number of publications. (**B**) Distribution of publications by corresponding authors’ countries and their international collaboration. TIS, Temporal Interference Stimulation; SCP, Single-Country Publication; MCP, Multiple-Country Publication.

**Figure 2:**
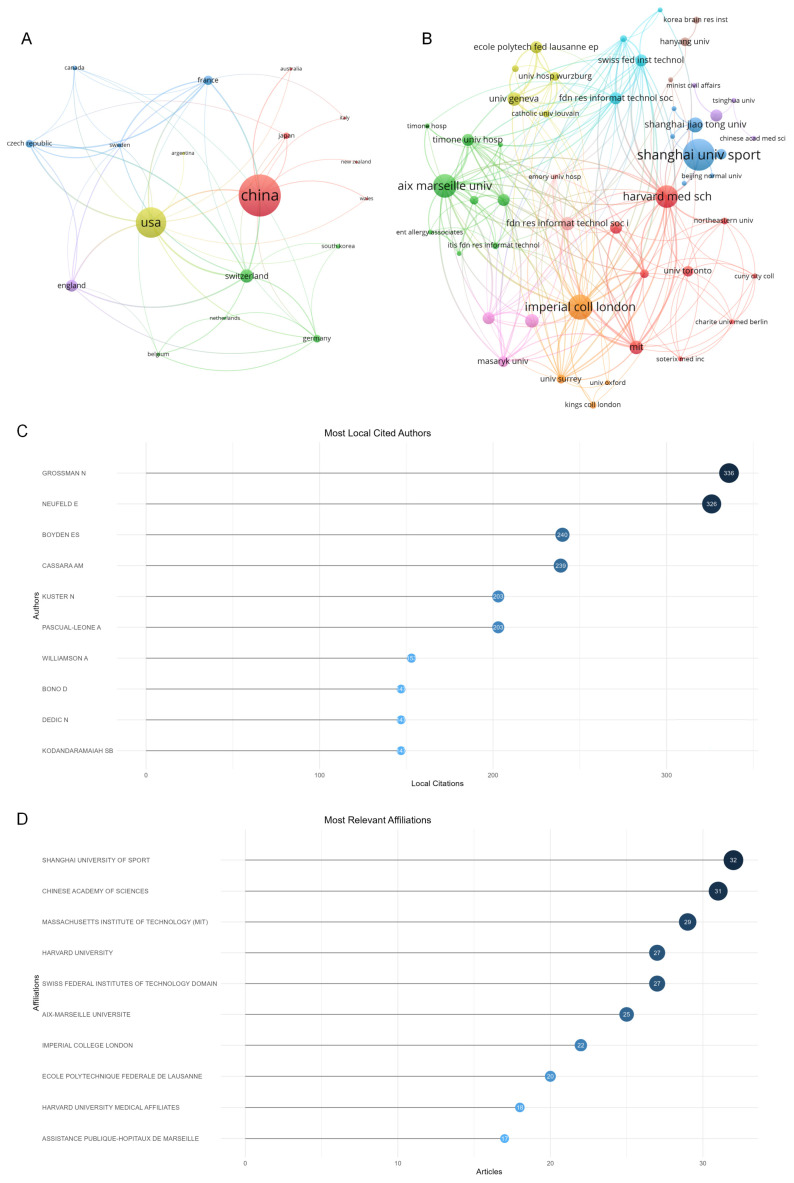
Collaboration networks and leading contributors in temporal interference stimulation research. (**A**) Country/region co-authorship network. (**B**) Institutional co-authorship network. In panels A and B, node size reflects publication output, links represent collaborative relationships, thicker links indicate stronger collaboration, and colors denote network clusters identified by VOSviewer. (**C**) The ten most locally cited authors, ranked by the number of citations received from other publications within the included dataset. (**D**) The ten most relevant affiliations, ranked by their associated number of publications. TIS, temporal interference stimulation.

A total of 181 unique records were retrieved from the WoSCC database for this analysis, with no relevant publications identified before 2017. The field of TIS was initiated by a seminal 2017 study [20] that introduced a novel, non-invasive approach for stimulating deep brain neurons through the application of multiple high-frequency electric fields that differed slightly in frequency. This technique was demonstrated to effectively activate hippocampal neurons in living mice without engaging neurons in the overlying cortex. As shown in Figure 1A, TIS research remains a nascent yet rapidly expanding field. After its emergence in 2017, the field underwent a brief exploratory phase until 2019, followed by a sharp and sustained increase in publication output, reaching a peak of 47 articles in 2024. As of 1 October 2025, 45 papers had already been published, suggesting that the upward growth trend is likely to continue. This trajectory highlights the burgeoning and enduring research interest in TIS over recent years. In terms of geographical distribution, analysis of corresponding authors’ countries revealed that China was the leading contributor, producing 72 publications (39.8% of total output), followed by the United States (34, 18.8%), the United Kingdom (12, 6.6%), Switzerland (9, 5.0%), and Korea (7, 3.9%). As shown in Figure 1B and detailed in Table 1, these nations were the most productive in this domain. The data also revealed a strong pattern of international collaboration. Countries such as the Czech Republic and France exhibited the highest collaboration rates, with all their papers being Multi-country Publications (MCPs), followed by Switzerland (77.8%) and Germany (75%). In contrast, China, the most prolific country, showed a relatively low collaboration rate (19.4%), whereas the United States maintained a high rate (47.1%). These collaborative relationships are visualized in the co-authorship network (Figure 2A), illustrating a global research landscape dominated by China and the United States as central hubs. At the author level, Grossman N was the most locally cited researcher within the included literature, with 336 local citations, followed by Neufeld E (*n* = 326) and Boyden ES (*n* = 240) (Figure 2C). Regarding institutional productivity, Shanghai University of Sport ranked first with 32 publications, followed by the Chinese Academy of Sciences (*n* = 31) and the Massachusetts Institute of Technology (*n* = 29) (Figure 2D).

### 3.2. Journal Analysis

**Figure 3:**
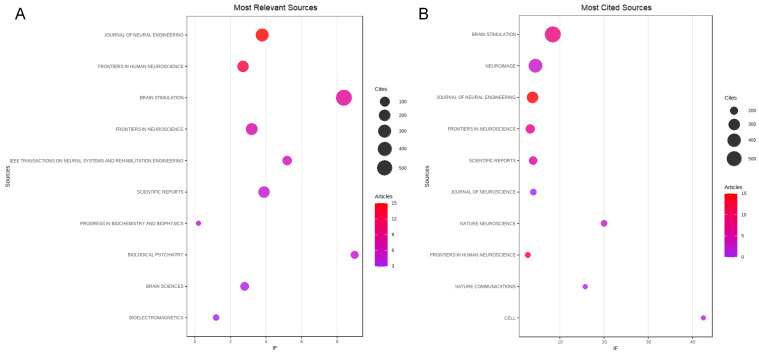
Analysis of academic journals. (**A**) The top 10 most relevant journals ranked by the number of publications. Bubble size indicates the number of citations, and color represents the number of articles. (**B**) The top 10 most cited journals. Bubble size indicates the number of citations, and the x-axis denotes the IF. IF, Impact Factor.

**Figure 4:**
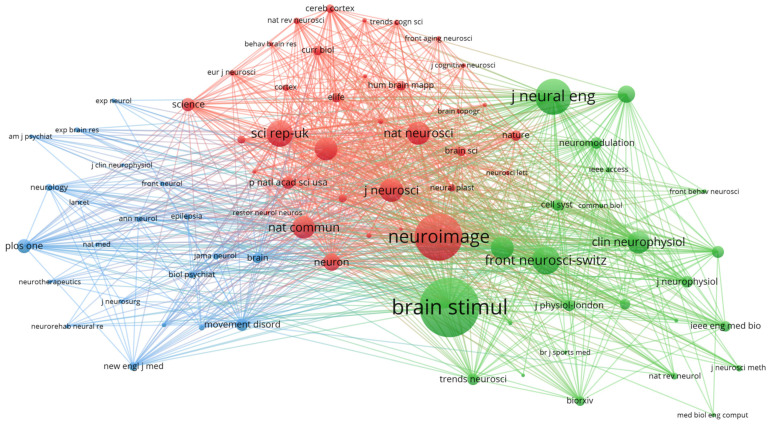
The co-citation network map of journals. The size of each node indicates the citation frequency of the corresponding journal, the connecting lines represent co-citation relationships, and the colors distinguish different clusters.

To identify the most influential journals in the field of TIS, we analyzed both publication volume and citation counts. As illustrated in Table 2 and depicted in Figure 3A, *the Journal of Neural Engineering* emerged as the leading publisher with 15 articles. It was followed by *Frontiers in Human Neuroscience* (*n* = 13), *Brain Stimulation* (*n* = 8), and *Frontiers in Neuroscience* (*n* = 7). These journals represent the most active platforms for publishing TIS research. Table 3 and Figure 3B showcase the most frequently cited journals in the field. *Brain Stimulation* was the most cited journal with 566 citations, followed by *NeuroImage* (430 citations), *the Journal of Neural Engineering* (307 citations), and *Frontiers in Neuroscience* (227 citations). It is noteworthy that top-tier general science and neuroscience journals, such as *NeuroImage*, *Nature Neuroscience*, and *Cell*, appear among the most cited sources despite having few or no primary articles in our dataset. This indicates that foundational research from outside the core brain stimulation literature significantly influences this emerging field. The interconnections and influence of these journals are further visualized in the journal co-citation network presented in Figure 4, which highlights central hubs like *Brain Stimulation*, *NeuroImage*, and *the Journal of Neural Engineering*.

### 3.3. Citation Bursts

**Figure 5:**
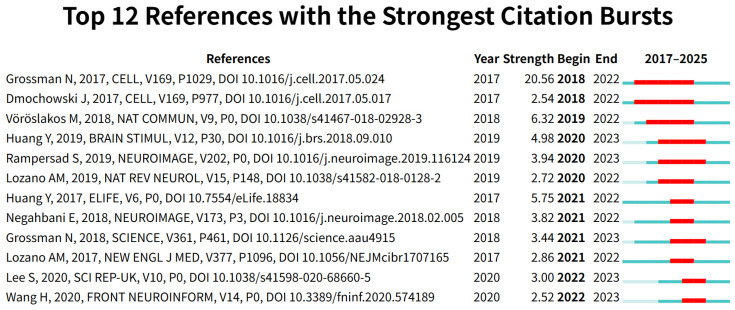
The top 12 references with the strongest citation bursts [3,20,23,30,31,32,33,34,35,36,37,38]. The red bars indicate the duration of the citation burst, from the beginning year to the end year. “Strength” indicates the intensity of the burst.

To delve deeper into the field’s core literature and research frontiers, we utilized CiteSpace to identify the top 12 references with the strongest citation bursts (Figure 5). The three references exhibiting the most pronounced bursts were as follows: (1) The seminal 2017 *Cell* paper, with an exceptionally high burst strength of 20.56 [20]; (2) a 2018 *Nature Communications study* (strength: 6.32) [30]; and (3) a 2017 *eLife* paper (strength: 5.75) [31]. The foundational 2017 paper introduced the concept of TIS, demonstrating its ability to non-invasively stimulate deep brain structures such as the mouse hippocampus [20]. This breakthrough work, which also showed the technique’s steerability, immediately generated significant interest, framing TIS as a potential noninvasive alternative to DBS. The other high-strength bursts [30,31] provided critical biophysical context for the entire field of transcranial electrical stimulation. They offered the first direct in vivo and ex vivo measurements of the electric fields generated within the rodent and human brain from scalp-applied currents, establishing that approximately 75% of the current is attenuated by the skull and soft tissues and that a field strength of at least ~1 V/m is likely necessary to directly affect neuronal spiking [30,31]. Furthermore, the citation burst timeline suggests that more recent bursts within the observation window were mainly associated with computational modeling and the practical implementation of TIS. Two key 2019 studies [32,33] were the first to extensively model TIS in realistic human heads. Their findings suggested that while suprathreshold stimulation as seen in mice is unlikely in humans at safe current levels, TIS may offer advantages over conventional multi-electrode tACS in terms of focality and steerability [32,33]. Subsequent 2020 studies further suggest an increasing emphasis on technical refinement, including the development of dedicated hardware with impedance monitoring and customized optimization pipelines to improve the precision of targeting individual brain structures [34,35].

Overall, the citation burst analysis highlights a clear research trajectory. It begins with the foundational concept of TIS in animal models, followed by a critical phase of contextualization through biophysical measurements and computational feasibility studies in humans. The current frontier is characterized by research comparing TIS to other multi-electrode approaches and developing methods for precise, individualized targeting. It is worth noting that Figure 5 ranks references by burst strength rather than publication recency. The predominance of earlier works likely reflects their longer citation accumulation windows. In contrast, recent publications may not have had sufficient time to develop detectable citation bursts, partly due to the data collection cutoff. Therefore, the absence of newly published papers from the top 12 is likely a consequence of inherent citation lag rather than limited scientific importance.

### 3.4. Keyword Clusters and Trend Topics

**Figure 6:**
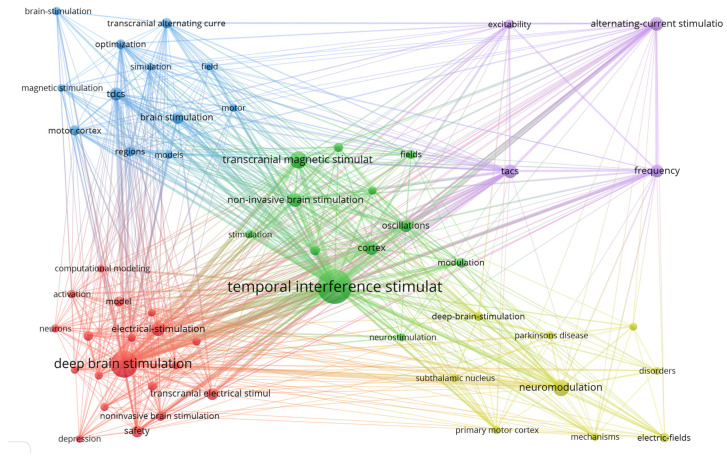
Keyword co-occurrence network map. Node size is proportional to keyword frequency, and colors indicate different thematic clusters identified by VOSviewer.

Following targeted thesaurus-based standardization of core TIS-related terms, including variants referring to “temporal interference” and “temporally interfering”, keywords occurring at least five times were included in the co-occurrence analysis. Table 4 presents the top 20 keywords, highlighting the principal research interests in the field. “Temporal interference stimulation” was the most frequent standardized term (*n* = 84), followed by “deep brain stimulation” (*n* = 54), “transcranial magnetic stimulation” (*n* = 22), and “neuromodulation” (*n* = 21). These results indicate that the literature is centered on the core TIS technique, its positioning within the broader neuromodulation landscape, and its potential application to deep brain targets. The keyword co-occurrence network further visualizes the thematic relationships among these frequently occurring terms and shows several clusters related to TIS, neuromodulation techniques, electric-field mechanisms, and disease-oriented applications (Figure 6).

**Figure 7:**
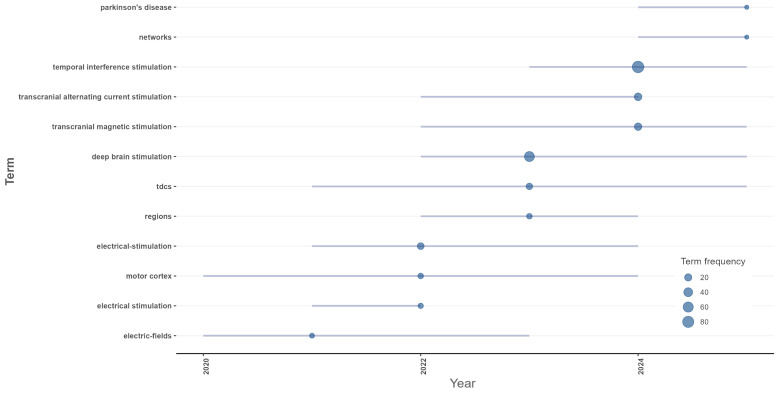
Trending topics in TIS research. Bubble position indicates the median publication year, horizontal lines indicate the interquartile range, and bubble size indicates term frequency. TIS, Temporal Interference Stimulation.

The trend-topic analysis further illustrated the temporal shift in research emphasis (Figure 7). Earlier topics were primarily related to electric fields, electrical stimulation, and the motor cortex, reflecting the field’s initial focus on biophysical principles and stimulation feasibility. Subsequent research increasingly emphasized deep brain stimulation, tDCS, temporal interference stimulation, and transcranial alternating current stimulation. More recent terms, including “networks” and “Parkinson’s disease”, suggest growing interest in network-level effects and disease-oriented applications. Nevertheless, these temporal patterns primarily reflect changes in research activity, keyword usage, and thematic attention. They should not be interpreted as definitive evidence of clinical efficacy, clinical maturity, or scientific consensus regarding the mechanisms and therapeutic value of TIS.

### 3.5. Comprehensive Analysis of Hotspots

Based on a comprehensive analysis of publication trends, influential journals, citation bursts, standardized keyword co-occurrence, and trend-topic patterns, we identified three primary research hotspots in the field of TIS: (1) Foundational Principles and Biophysical Modeling: Originating from the seminal 2017 work [20], this hotspot uses computational modeling to explore the underlying physics of TIS. Such simulations are critical for assessing the feasibility, safety, and biophysical properties of TIS-induced electric fields in realistic human head models [34,39,40]. (2) Comparative Efficacy and Positioning: This area evaluates the advantages of TIS over other neuromodulation techniques, focusing on its capacity for non-invasive deep brain stimulation with enhanced focality and steerability [41,42]. (3) Application, Targeting, and Optimization: This hotspot is dedicated to exploring the potential of translating TIS into clinical and research applications. This involves early attempts to demonstrate its steerability in activating functional networks [43,44], devising precise targeting strategies for deep brain structures [42,45], and engineering custom hardware and software pipelines for improved accuracy and real-time monitoring [46,47].

## 4. Discussion

This study provides a comprehensive bibliometric analysis of the rapidly emerging field of TIS, characterizing the growth in publication output since 2017 and mapping its global research landscape, knowledge structure, and developmental trajectories. Recent scoping and narrative reviews have provided valuable qualitative syntheses of the physiological mechanisms, parameter optimization, and clinical efficacy of TIS, but these studies have primarily focused on selected experimental evidence. In contrast, the present bibliometric analysis offers a quantitative macroscopic perspective based on the available TIS literature indexed in WoSCC. By applying bibliometric and visualization methods, this study complements previous reviews by characterizing collaboration networks, tracing temporal changes in influential references through citation burst analysis, and identifying major thematic clusters and emerging research directions.

### 4.1. General Information

Annual publication trends (Figure 1A) show that since the first report in 2017, TIS has undergone rapid growth, reflecting increasing academic interest and recognition as a novel neuromodulation technique. China and the United States are the leading contributors (Figure 1B, Table 1), serving as the main global research hubs. The collaboration network (Figure 2A) highlights strong interconnections among European countries such as Switzerland, Germany, and France, whereas China’s relatively low rate of multi-country publications suggests a more independent research approach. Journal analysis identifies the *Journal of Neural Engineering* and *Frontiers in Human Neuroscience* as the main outlets for TIS studies (Figure 3A, Table 2). In contrast, *Brain Stimulation* and *NeuroImage*, along with broad-scope journals like *Cell* and *Nature Neuroscience*, are the most influential sources based on citations (Figure 3B, Table 3). The journal co-citation network (Figure 4) further confirms their central role in shaping the TIS knowledge base. Furthermore, the literature landscape reveals a strong citation concentration around a small number of pioneering research groups. Because TIS is a recently emerged and technically complex field, initial breakthroughs in biophysical validation and computational head modeling have been predominantly driven by a few specialized laboratories. This concentration accurately reflects the current developmental structure of the discipline. These core groups have established the foundational knowledge base that is now guiding broader international investigations.

### 4.2. Hotspots and Development Trends

Analysis of keywords, citation bursts, and thematic evolution revealed three main research hotspots in TIS: biophysical mechanisms, comparison with other neuromodulation techniques, and application to specific brain targets. These themes outline the field’s developmental framework.

Research into the fundamental biophysical principles of TIS is a core theme in the field. In the 2017 seminal study, the core principle of TIS was built on two basic biophysical premises: that high-frequency (kHz-level) alternating electric fields can penetrate the skull and brain tissue with minimal attenuation [20,48], and that neuronal membranes are insensitive to high-frequency signals but responsive to low-frequency (Hz-level) signals. One focus has been the use of computational modeling to understand and predict the behavior of TIS-induced electric fields. In addition, direct in vivo measurements have provided important evidence on electric field attenuation through the skull and soft tissues [30,31]. Regarding the mechanism of suprathreshold neural activation, two main hypotheses have emerged. The “envelope demodulation hypothesis” posits that neurons actively rectify the low-frequency signal through nonlinear ion channel dynamics [49], whereas the “linear integration hypothesis” proposes that TIS is essentially a spatially focused kilohertz-frequency stimulation [50]. These two theories may be complementary rather than conflicting [24], though further investigation is required.

Based on the high co-occurrence of TIS with terms like “transcranial magnetic stimulation” and “tDCS”, positioning TIS within the existing landscape of neuromodulation technologies appears to be a significant research direction. Conventional tES produces diffuse and superficial electric fields because of current shunting in the skull and soft tissues [51,52]. Similarly, TMS struggles to penetrate deeply without sacrificing focality [15,53] and its reach remains largely limited to cortical regions [54]. In contrast, TIS theoretically achieves precise modulation of deep targets. It forms a low-frequency envelope deep within the brain through the temporal interference of two high-frequency electric fields. This mechanism avoids direct activation of the overlying cortex. Compared to TFU, TIS is less constrained by the skull’s acoustic impedance and thermal effects [55]. Importantly, TIS is positioned as a non-surgical alternative to DBS, offering a lower-risk and more accessible therapeutic option [56]. Computational simulations in realistic human head models have demonstrated the potential of TIS [32,33]. These influential studies indicate that TIS may offer significant advantages over traditional multi-electrode tACS. Specifically, it enhances focality and allows researchers to non-invasively steer stimulation to the target.

Recent research hotspots have shifted towards targeted optimization and practical applications. As indicated by recent citation bursts (Figure 5), two 2020 studies concentrate on developing optimized, individualized stimulation protocols and creating dedicated hardware with real-time monitoring capabilities [34,35]. Findings from fMRI studies suggest that TIS may modulate brain networks rather than just individual regions [57,58], mirroring evidence from rTMS and DBS studies [59]. Studies in healthy volunteers have found that TIS can locally and non-invasively modulate hippocampal activity and improve memory performance [56,60]. Furthermore, small-scale preliminary studies have revealed the potential of TIS in intervening in Parkinson’s disease [61] and schizophrenia [62,63]. However, it is crucial to interpret these findings with caution, as the current clinical evidence remains preliminary and requires extensive validation in larger cohorts to address the inconsistencies observed in recent human trials. Future research may involve larger, better-designed trials employing novel methods such as phase-modulated interference (PMI) and multi-point temporal interference (MTI) [64], as well as individualized approaches like closed-loop stimulation and advanced modeling [34,65].

### 4.3. Challenges and Future Directions

TIS faces both skepticism and challenges, one of which is the unavoidable phenomenon of High-Frequency Conduction Block (CB) [49]. CB occurs when nerve fibers are continuously exposed to a high-frequency electric field of sufficient intensity. This persistent depolarization causes their sodium ion channels to enter an inactivated state [66,67]. This challenges the fundamental premise of TIS selectivity, as it may achieve desired neural activation in the target region while causing unintended, widespread conduction block along the path to the target [66]. This also raises safety concerns about TIS due to its potential for unpredictable off-target effects on the cortex [68]. Consequently, a consensus is forming that future animal and human trials should include control and measurement methods to detect CB effects, rather than focusing solely on whether the target area is successfully activated [49]. Awareness of the CB risk has also spurred the development of more sophisticated TIS techniques aimed at reducing field strength in off-target areas, such as multipolar TIS and phase-canceling TIS [47,69].

Although our thematic evolution analysis shows a growing academic focus on human applications, broader domain knowledge emphasizes that TIS technology faces the scaling problem when transitioning from small animal models to clinical trials [70]. Unlike in mouse models, where suprathreshold stimulation of the hippocampus can be achieved with a total current of 0.776 mA [33], achieving the same effect in humans would require currents exceeding safety limits [24]. Therefore, the mechanism of TIS in humans, compared to the direct stimulation of DBS, may be better characterized as that of a subthreshold modulator [71]. Future research must thus fully account for individual differences in anatomical structure and brain state [34].

The results of TIS application in humans are currently inconsistent. For example, while one study reported that TIS applied to the striatum improved motor skill learning [72], another study found that it impaired motor learning, possibly due to different stimulation patterns [73]. Some studies have failed to observe clear central effects of TIS [66,74]. Existing studies on patients are preliminary and small in scale [61,63]. This calls for future large-sample, high-quality clinical research.

Although interest in clinical applications is growing, significant methodological gaps remain. The current evidence base predominantly consists of small-scale, single-session studies involving healthy volunteers. There is a distinct shortage of adequately powered, sham-controlled trials in patient populations. Future research must prioritize rigorous randomized controlled trials in specific clinical cohorts to determine clinically meaningful therapeutic endpoints.

### 4.4. Limitations

This study has several limitations. First, the formal bibliometric analyses were based solely on the WoSCC database. Although WoSCC provides standardized bibliographic metadata and structured cited-reference information suitable for co-citation, citation burst, collaboration network, and keyword analyses, the exclusion of other databases such as Scopus and PubMed may have influenced the coverage of the included literature. These databases may index additional journals, conference proceedings, or records not covered by WoSCC, which could affect publication counts and the relative rankings of countries, institutions, journals, and authors. Therefore, the present findings should be interpreted as reflecting the WoSCC-indexed landscape of TIS research rather than an exhaustive representation of all TIS-related publications across databases. Database coverage and citation indicators are source-dependent, and the resulting bibliometric rankings should be interpreted within this context. Moreover, bibliometric analysis inherently cannot evaluate the methodological quality, evidentiary strength, or clinical validity of individual studies. Publication counts, citation bursts, keyword co-occurrence, and trend-topic patterns reflect research activity and scholarly visibility rather than definitive evidence of clinical efficacy or scientific consensus. Citation-based indicators are also time-dependent. Recent high-quality papers may receive fewer citations simply because of their publication date, failing to fully reflect the immediate scientific impact of the most current research trends. These limitations should be considered when interpreting the bibliometric rankings and thematic patterns identified in this study.

## 5. Conclusions

TIS has rapidly emerged as an important research frontier in neuromodulation, with the potential to modulate deep brain structures non-invasively. This study outlines the field’s developmental trajectory and highlights key research directions, including elucidation of its biophysical mechanisms, comparison with other neuromodulation modalities, and optimization of stimulation strategies for experimental and potential clinical applications. Despite its theoretical advantages in depth and focality, substantial engineering, physiological, and methodological challenges remain. Addressing these barriers, particularly through individualized modeling and rigorously designed, sham-controlled studies in human participants and patient populations, will be essential for clarifying whether TIS can become a reliable clinical intervention.

## Figures and Tables

**Table 1:** Most relevant countries by corresponding authors.

Country	Articles	Articles_Ratio	SCP	MCP	MCPs_Ratio
China	72	39.8	58	14	19.4
USA	34	18.8	18	16	47.1
United Kingdom	12	6.6	5	7	58.3
Switzerland	9	5	2	7	77.8
Korea	7	3.9	6	1	14.3
Canada	6	3.3	5	1	16.7
Iran	5	2.8	5	0	0
Japan	5	2.8	5	0	0
Czech Republic	4	2.2	0	4	100
Germany	4	2.2	1	3	75
France	3	1.7	0	3	100
Netherlands	3	1.7	1	2	66.7
Argentina	2	1.1	1	1	50
Belgium	1	0.6	1	0	0
Finland	1	0.6	0	1	100
Greece	1	0.6	1	0	0
Ireland	1	0.6	1	0	0
New Zealand	1	0.6	1	0	0
Norway	1	0.6	1	0	0
Spain	1	0.6	1	0	0

Note. MCP, Multiple-Country Publication; SCP, Single-Country Publication.

**Table 2:** Top 10 journals with the most publications.

Sources	Documents	Cites	IF
*Journal of Neural Engineering*	15	307	3.8
*Frontiers in Human Neuroscience*	13	178	2.7
*Brain Stimulation*	8	566	8.4
*Frontiers in Neuroscience*	7	227	3.2
*IEEE Transactions on Neural Systems and Rehabilitation Engineering*	6	83	5.2
*Biological Psychiatry*	5	45	9
*Progress in Biochemistry and Biophysics*	5	2	0.2
*Scientific Reports*	5	211	3.9
*Brain Sciences*	4	63	2.8
*Bioelectromagnetics*	3	13	1.2

**Table 3:** Top 10 most cited journals.

Sources	Cites	Documents	IF
*Brain Stimulation*	566	8	8.4
*NeuroImage*	430	3	4.5
*Journal of Neural Engineering*	307	15	3.8
*Frontiers in Neuroscience*	227	7	3.2
*Scientific Reports*	211	5	3.9
*Journal of Neuroscience*	183	0	4
*Nature Neuroscience*	181	3	20
*Frontiers in Human Neuroscience*	178	13	2.7
*Cell*	176	1	42.5
*Nature Communications*	176	1	15.7

**Table 4:** The top 20 keywords based on co-occurrence analysis.

Rank	Keywords	Count
1	Temporal Interference Stimulation	84
2	Deep Brain Stimulation	54
3	Transcranial Magnetic Stimulation	22
4	Neuromodulation	21
5	Cortex	16
6	Electrical-Stimulation	16
7	tACS	15
8	Non-Invasive Brain Stimulation	15
9	Alternating-Current Stimulation	15
10	tDCS	14
11	Frequency	13
12	Oscillations	12
13	Safety	11
14	Brain Stimulation	11
15	Transcranial Electrical Stimulation	11
16	Regions	9
17	Transcranial Alternating Current Stimulation	9
18	Motor Cortex	9
19	Optimization	8
20	Noninvasive Brain Stimulation	8

## Data Availability

The datasets and code used and/or analysed during the current study are available from the corresponding authors on reasonable request.
